# Microfracture technique versus osteochondral autologous transplantation mosaicplasty in patients with articular chondral lesions of the knee: a prospective randomized trial with long-term follow-up

**DOI:** 10.1007/s00167-014-2843-6

**Published:** 2014-01-21

**Authors:** Svend Ulstein, Asbjørn Årøen, Jan Harald Røtterud, Sverre Løken, Lars Engebretsen, Stig Heir

**Affiliations:** 1 Department of Orthopedic Surgery, Akershus University Hospital, 1478 Lørenskog, Norway; 2 Oslo Sports Trauma Research Center, Oslo, Norway; 3 Institute of Clinical Medicine, Akershus University Hospital, University of Oslo, Lørenskog, Norway; 4 Department of Orthopaedics, Oslo University Hospital, Oslo, Norway; 5 Norwegian Knee Ligament Registry, Bergen, Norway; 6 Department of Orthopaedic Surgery, Martina Hansens Hospital, Bærum, Norway

**Keywords:** Chondral lesion, Microfracture, Mosaicplasty, Long-term follow-up, Lysholm, KOOS

## Abstract

**Purpose:**

To compare long-term functional and radiological outcome following microfracture technique (MF) versus osteochondral autologous transplantation (OAT) mosaicplasty for treating focal chondral lesions of the knee.

**Methods:**

Twenty-five patients (mean age 32.3 years, SD 7.7) with a full-thickness (International Cartilage Repair Society grade 3 or 4) chondral lesion of the articulating surface of the femur were randomized to either MF (*n* = 11) or OAT mosaicplasty (*n* = 14). At a median follow-up of 9.8 years (range 4.9–11.4), the patients were evaluated using Lysholm score (*n* = 25), Knee Injury and Osteoarthritis Outcome Score (KOOS, *n* = 25), isokinetic quadriceps measurement and hamstring strength measurement (*n* = 22) and standing radiographs (*n* = 23).

**Results:**

There were no significant differences in Lysholm score, KOOS, isokinetic muscle strength or radiographic osteoarthritis between MF-treated patients and OAT mosaicplasty-treated patients at follow-up. Mean Lysholm score at follow-up was 69.7 [95 % confidence interval (CI), 55.1–84.4] for the MF group and 62.6 (95 % CI, 52.6–72.6) for the OAT mosaicplasty group.

**Conclusion:**

At long-term follow-up, there were no significant differences between patients treated with MF and patients treated with OAT mosaicplasty in patient-reported outcomes, muscle strength or radiological outcome.

**Level of evidence:**

Therapeutic study, Level II.

## Introduction

Chondral or osteochondral lesions of the knee eligible for cartilage repair surgery are diagnosed in 5–10 % of all knees subjected to knee arthroscopy [1, 20] and may contribute to disability and premature osteoarthritis (OA) [29]. Furthermore, focal chondral lesions of the knee have been shown to impair quality of life similar to patients scheduled for knee replacement, even though the chondral lesion patients are 30 years younger [18].

Various cartilage repair techniques have been developed. Resurfacing techniques include abrasion arthroplasty [24], Pridie drilling [36] and microfracture technique (MF) [3, 43]. MF procedures stimulate and recruit mesenchymal cells from the subchondral bone marrow and subsequently form a fibrin clot that eventually turns into a predominantly fibrocartilaginous regenerate with inferior biomechanical characteristics compared to native hyaline articular cartilage [11]. Despite fibrocartilage formation, several short- to mid-term follow-up studies following MF treatment of chondral lesions report significant pain relief and improvement in knee function [32, 33, 43].

Grafting and transplantation procedures, like autologous chondrocyte implantation (ACI) [6] and osteochondral autologous transplantation (OAT) mosaicplasty [16] gained popularity after introduction in the 1990s. The OAT mosaicplasty technique involves open or arthroscopic transplantation of multiple cylindrical osteochondral grafts from the relatively less weight-bearing periphery of the articular surface to the cartilage defect, thus providing a hyalinecartilage-covered resurfacing [2, 22]. Case series and comparative trials have reported 83–92 % good to excellent short- to mid-term results following OAT mosaicplasty [8, 13, 15]. Even though MF and OAT mosaicplasty have proven to be effective in short- to mid-term follow-up studies, knowledge regarding long-term outcome remains uncertain [4, 8, 14, 32, 41, 42]. To our knowledge, there is only one prospective randomized study comparing the long-term outcomes following MF and OAT mosaicplasty [12]. Due to the limited information on the long-term outcome after these two common cartilage repair techniques, patient information and decision-making regarding treatment options is challenging for the orthopaedic surgeon.

In the present prospective randomized study, the purpose was to compare long-term functional and radiological outcome following MF and OAT mosaicplasty for full-thickness chondral lesions of the knee. The null hypothesis was that there is no difference in patient-reported outcomes or radiographic OA between MF-treated patients and OAT mosaicplasty-treated patients at long-term follow-up.

## Materials and methods

Twenty-five patients [mean age 32.3 years, standard deviation (SD) 7.7] were enroled in the study between November 2000 and June 2006. Three orthopaedic cartilage repair centres participated in the study, and experienced knee surgeons performed both the selection of the patients and the surgical procedure. Informed consent was obtained from all patients.

Inclusion criteria were an arthroscopically verified chondral or osteochondral lesion of International Cartilage Repair Society (ICRS) grade 3 or 4 [7] located on the femoral condyle or trochlea, with an area between 2 and 6 cm^2^ and depth <10 mm. Additionally, the patients had to be 18–50 years of age with Lysholm score <80 and Tegner score <6.

Exclusion criteria were radiographic osteoarthritis (OA), major malalignment, major ligament injury or instability, extension deficit >3°, flexion deficit >5° and chondral lesion(s) of ICRS grade 3 or 4 on the tibial plateau or patella. Patients were also excluded if they had contralateral impaired knee function that might influence the ability to follow the rehabilitation protocol.

Randomization between MF and OAT mosaicplasty was performed in the operating room, following arthroscopic debridement. Patients were randomized by a restricted shuffled approach [39] in blocks of 10, allocation ratio 1:1, using sequentially numbered sealed envelopes to assign treatment. The block randomization approach used ensured that all centres/surgeons performed both procedures and also ensured randomization to surgeon. Twenty-five patients were included, and in accordance with randomization, 14 patients were treated with OAT mosaicplasty and 11 patients with MF. Group characteristics at inclusion are shown in Table 1.

**Table 1:** Characteristics of the study groups at inclusion

	MF (*n* = 11)	OAT Mosaicplasty (*n* = 14)
Age, years^a^ (*n* = 25)	31.7 (8.0)	32.7 (7.8)
Duration of symptoms, mos^a^ (*n* = 24)	111.0 (77.3)	75.8 (73.5)
Gender, *n* (%)		
Females	5 (45)	6 (43)
Males	6 (55)	8 (57)
Right/left	7/4	8/6
Lesion localization (*n* = 25)		
Trochlea	0	2
Medial femoral condyle	10	10
Lateral femoral condyle	1	2
Lesion size^b^ (*n* = 25)	2.6 (2.0–5.2)	3.0 (2.0–6.0)
Injury mechanism (*n* = 25)		
Gradual onset	0	4
Trauma/acute onset	5	6
Osteochondritis dissecans	6	4
ICRS classification (grade 3/4)	4/7	8/6
Previous cartilage surgery^c^	3	1
Tegner activity level score^b^ (*n* = 23)	3 (0–4)	2.5 (0–4)

*ICRS* International Cartilage Repair Society

^a^Mean and (standard deviation)

^b^median and (range)

^c^Resurfacing and/or grafting and/or transplantation

A total of 19 patients were excluded from the study. In most cases, this was due to findings during the arthroscopic assessment, e.g. size or localization of the chondral lesion not in accordance with the inclusion criteria or additional ICRS grade 3 or 4 chondral lesions of the tibia or patella. Two patients declined surgery due to pregnancy, and two patients withdrew their consent at the time of surgery as they insisted on being treated with one of the surgical techniques.

## Treatment

### Microfracture technique

The procedure was arthroscopic and the principles of the technique introduced by Steadman et al. [43] were used. Debridement of all damaged and unstable cartilage was performed, as to obtain stable and healthy cartilage edges. An arthroscopic awl (Linvatec) was then used to perform multiple holes (“microfractures”) about 3–4 mm apart. The depth of the holes was considered appropriate when “fat-pearls” emerged from the subchondral bone.

### OAT mosaicplasty

Following application of a tourniquet, the OAT mosaicplasty was performed through a medial parapatellar arthrotomy or a mini-invasive arthrotomy, depending on the lesion size and localization. Debridement was done similar to that described for MF. The OAT mosaicplasty procedure was performed as described by Hangody et al. [16] by obtaining small cylindrical osteochondral grafts (3.5, 4.5 or 6.6 mm in diameter) from the minimal weight-bearing periphery of the femoral condyles and transplanting them “press-fit” to recipient tunnels in the prepared lesion site (Acufex^®^, Smith&Nephew). At the end of the procedure, the knee was moved through a full range of motion to check the stability of the osteochondral plugs.

For both techniques, one dose of prophylactic antibiotics was administrated intravenous in advance of the procedure, followed by two dosages postoperatively. Intra-articular Bupivacaine (Marcain^®^) was installed at the end of the procedure.

### Postoperative care

All patients were hospitalized for a minimum of 5 days. Continuous passive motion (Kinetec^®^) 3–4 h × 2/day was started the first postoperative day and continued for four days. Cold therapy and compression (Aircast Knee Cryo/Cuff^®^) were applied the two first days postoperatively to reduce swelling and pain.

### Rehabilitation

The rehabilitation programme was similar for both groups. The programme used was based on the principles and recommendations of Hangody and Steadman [17, 43]. A maximum load of 15–20 kg weight bearing was allowed the initial 6 weeks postoperatively, following gradually discontinuing of the crutches up to 8 weeks. From 8 weeks, progression to full weight bearing was encouraged. Physiotherapist-guided rehabilitation was initialized immediately postoperatively and was continued for a minimum of 6 months. The rehabilitation programme included exercises aiming to restore full range of motion and proprioceptive neuromuscular control as soon as possible, progressing to dynamic strength exercises from 6 weeks postoperatively. Patients were generally allowed return to full activity after 6 months. However, participation in competitive contact sports or other activities that may expose the knee to pivoting forces was discouraged until 12 months postoperatively.

## Outcome measures

All outcome measures were obtained both at baseline and follow-up, except for isokinetic muscle strength measurements, which were performed only at follow-up. In addition to the outcome measures, all patients were also questioned about any additional surgical procedures to the knee during the follow-up period.

### Lysholm score

The primary outcome measure was the Lysholm score [44], which is an 8-item (limp, support, locking, instability, pain, swelling, stair climbing and squatting) questionnaire. The total score is the sum of each response to the 8 items, of a possible score of 100 (100 = no symptoms or disability) The Lysholm score is validated for patients with cartilage injuries [26], and age and gender-specific population-based reference data have been established [5]. At follow-up, the Lysholm questionnaire was completed by the patients prior to the examination [21].

### The Knee Injury and Osteoarthritis Outcome Score (KOOS)

The KOOS is a self-reported assessment tool consisting of 42 questions distributed between 5 separately scored subscales: pain, other symptoms, activities of daily living (ADL), function in sport and recreation (Sport/Rec) and knee-related quality of life (QoL). Each subscale score is converted to a 0 (worst)–100 (best) scale. The KOOS is considered as a valid, reliable and responsive questionnaire for patients with chondral lesions of the knee [10, 38]. Age and gender-specific population-based reference data of the KOOS have been established [35]. A difference or change of 10 points or more in either of the subscales is considered as clinically relevant [10, 37]. At follow-up, the KOOS questionnaire was completed by the patients prior to the examination.

### Isokinetic muscle strength

Isokinetic quadriceps and hamstring muscle strength tests were performed at follow-up. It has previously been shown that muscular strength deficits in various knee disorders are associated with a poorer outcome, and two recently published studies found highly significant side-to-side differences in knee-related muscle strength in ACI-treated patients [27, 30]. In addition, since this is a comparative study between an arthroscopic and an open procedure, muscle strength assessments were considered relevant. Muscle strength was measured using a Biodex 6000 dynamometer (Biodex Medical System Inc., Shirley, New York). This device gives reliable and valid measurements of dynamic muscle function on variables related to torque, power and endurance [9]. Before testing, the patients did 10-min warm-up on a stationary bike. The test protocol consisted of five repetitions at an angular velocity of 60°/s in a concentric mode. Two physiotherapists, both blinded to the treatment, performed the measurements. Comparison was made between involved and uninvolved knee. The parameter used for analysis was peak torque/highest muscular force output (Nm) expressed as percentage deficit compared to the uninjured leg.

### Radiographs

Radiographs were performed in the AP-plane with the patients standing with semi-flexed knees. Evaluation and grading of anonymized radiographs were done according to the original Kellgren and Lawrence criteria [23] of knee OA (0 normal to 4 severe). The grading was done by three of the authors (SU, AÅ and SL) by consensus agreement.

The study was approved by the Regional Ethical Committee of South-Eastern Norway, University of Oslo, ID 155-00066.

### Statistical analysis

The sample size required to detect a difference in Lysholm score of 15 between groups was estimated by using the Altman nomogram. In addition to the predetermined power (0.80) and level of significance (0.05), the estimation is based on the calculation of the standardized difference, i.e. the difference in Lysholm score to be detected divided by the expected SD. Based on previous studies [40], the SD was expected to be 17, giving a standardized difference of 15/17 = 0.88. Using these figures, the Altman’s nomogram revealed that 20 patients in each treatment group would be sufficient.

SPSS software version 20 (Chicago, IL, USA, 2006) was used for statistical analysis. Lysholm, KOOS and isokinetic muscle strength deficits compared to uninjured leg at follow-up were compared between the treatment groups using Mann–Whitney *U* test. Changes in Lysholm and KOOS from baseline to follow-up were compared using Wilcoxon signed rank test. Changes in radiographic appearance according to Kellgren–Lawrence classification were compared between the two groups using Fishers exact test. Level of significance was defined as *p* ≤ 0.05.

## Results

At a median follow-up of 9.8 years (range 4.9–11.4 years), all patients (25/25) reported Lysholm score and KOOS. One patient had moved abroad, and another was not available for examination in the outpatient clinic. However, these patients were contacted by postal mail and telephone, and returned their questionnaires.

**Fig. 1:**
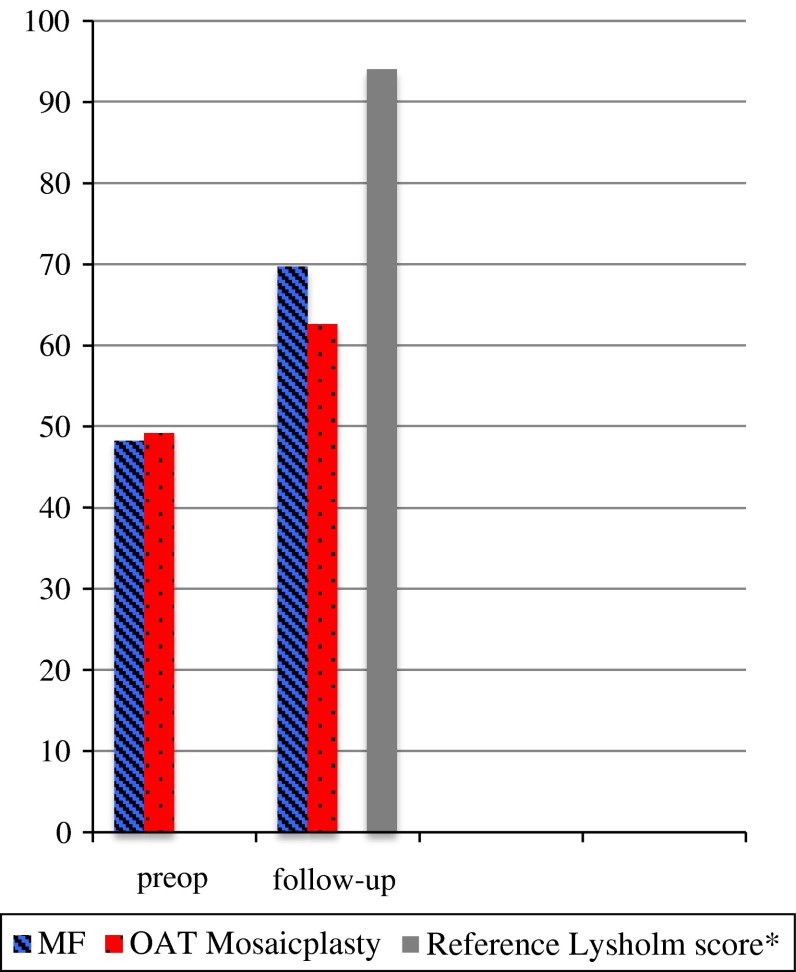
Mean Lysholm score for patients treated with MF (*n* = 11) and OAT mosaicplasty (*n* = 14) at preoperative and follow-up. ^*^Lysholm score acquired from a normal, healthy population as a standard point of reference for the injured or postsurgical knee, as described by Briggs, K.K. et al., Am J Sports Med, 2009

Mean Lysholm score for patients treated with MF and OAT mosaicplasty at baseline and at follow-up are shown in Fig. 1. No significant differences in mean Lysholm score were detected between MF-treated patients and OAT mosaicplasty-treated patients at follow-up (n.s.), or in mean change from baseline to follow-up (Table 2). MF-treated patients scored 48.2 (95 % CI, 38.2–58.2) preoperatively and OAT mosaicplasty-treated patients 49.2 (95 % CI, 43.0–55.4). The mean Lysholm score at follow-up in the MF group was 69.7 (95 % CI, 55.1–84.4) compared to 62.6 (95 % CI, 52.6–72.6) in the OAT mosaicplasty group. The increase in Lysholm score from baseline to follow-up was significant for both groups (Table 2).

**Table 2:** Mean change in Lysholm score and Knee Injury and Osteoarthritis Outcome Score from preoperative to follow-up, and mean difference in change over time between the MF group and OAT mosaicplasty group

	MF	OAT Mosaicplasty	MF vs OAT mosaicplasty	*p* value
	Change over time	Change over time	Change over time	
	Mean (95 % CI)	Mean (95 % CI)	Mean difference (95 % CI)	
Lysholm	21.6 (3.7–39.4)	13.4 (0.9–25.8)	8.2 (−11.7 to 28.1)	n.s
KOOS Pain	20.6 (2.8–38.3)	11.8 (−2.8 to 26.4)	8.8 (−12.7 to 30.3)	n.s
KOOS Symptoms	17.4 (2.6–32.2)	8.5 (−3.5 to 20.6)	8.9 (−8.9 to 26.7)	n.s
KOOS ADL	13.0 (−3.8 to 29.8)	7.5 (−4.3 to 19.3)	5.5 (−13.4 to 24.4)	n.s
KOOS Sport/Rec	32.4 (13.3–51.6)	41.3 (23.7–58.9)	−8.9 (−33.4 to 15.7)	n.s
KOOS QoL	34.6 (15.1–54.0)	25.0 (10.6–39.3)	9.6 (−12.7 to 31.9)	n.s

Change over time = follow-up minus preoperative

Mean difference = mean change over time in MF group minus mean change over time in OAT mosaicplasty group

*CI* confidence interval, *p* level of significance, *ADL* activities in daily living, *Sport/Rec* function in sport and recreation, *QoL* knee-related quality of life

**Fig. 2:**
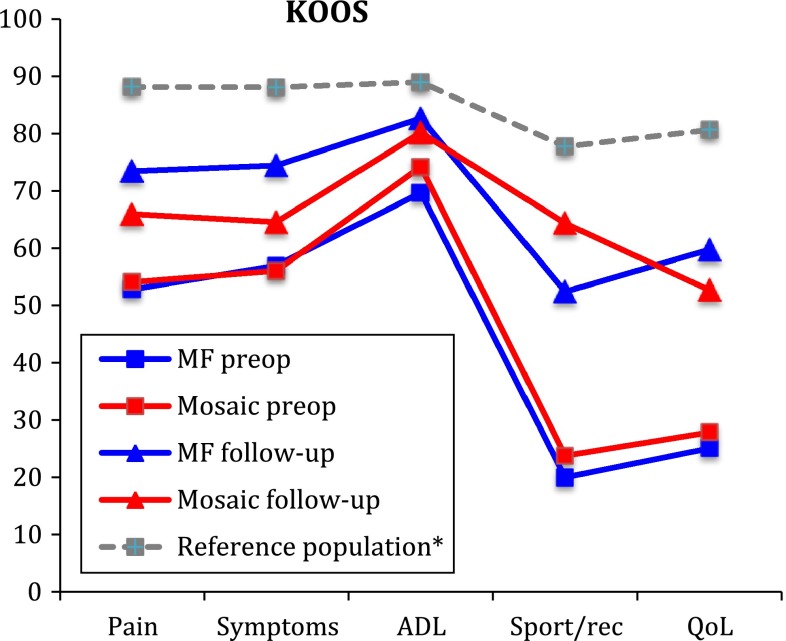
Knee Injury and Osteoarthritis Outcome Score (KOOS) at inclusion and follow-up for the MF group and the OAT mosaicplasty group. ^*^Reference population as described by Paradowski et al. [35] BMC Musculoskelet Disord

The KOOS profiles with mean scores at inclusion and at follow-up for the MF group and the OAT mosaicplasty group are shown in Fig. 2. There were no significant differences between the two groups in any of the KOOS subscales at follow-up or in the changes from baseline to follow-up (Table 2). The increase in KOOS from baseline to follow-up within the treatment groups was significant for all subscales except for ADL in the microfracture group, and pain, symptoms and ADL in the OAT mosaicplasty group (Table 2).

Isokinetic muscle strength measurements (*n* = 22) of the knee extensors and flexors at follow-up are shown in Table 3. There were no significant differences between the MF group and OAT mosaicplasty group in mean strength deficit of the affected knee. A significant mean extension strength deficit of the affected knee, compared to the unaffected, was detected in the OAT mosaicplasty group.

**Table 3:** Muscle strength measurements expressed as peak torque in Nm [mean (95 % CI)]

	MF (*n* = 10)			OAT mosaicplasty (*n* = 12)			*p* value
	Injured leg	Contralateral leg	% deficit	Injured leg	Contralateral leg	% deficit	
Extension peak torque 60°/s	159.4 (104.2–214.5)	179.3 (128.0–230.7)	7.1 (−22.1–36.3)	164.5 (132.8–196.1)	199.4 (165.4–233.5)	17.6 (8.9–26.3)	n.s
Flexion peak torque 60°/s	88.3 (57.1–119.4)	99.9 (73.6–126.1)	13.5 (−4.8–31.9)	92.7 (70.9–114.4)	98.9 (81.4–116.4)	6.7 (−7.3–20.7)	n.s

% Deficit = strength deficit of the operated knee in percentage, compared to contralateral leg

*p* values refer to Mann–Whitney nonparametric test used to analyse differences in strength deficit between the two treatment groups (MF vs. OAT mosaicplasty)

*n.s* non-significant

Twenty-three patients performed radiographic examination at follow-up. No patient had radiological signs of osteoarthritis of any knee at inclusion. Osteoarthritis was defined as Kellgren–Lawrence ≥2 and was detected in the affected knee in 5 of 11 patients in the MF group and 2 of 12 in the OAT mosaicplasty group at follow-up (*p* = 0.193). Osteoarthritis in the unaffected leg was detected in 3 of 11 knees in the MF group and in 1 of 12 knees in the OAT mosaicplasty group.

Mean body mass index (BMI) at follow-up was 28.2 (SD 4.2) for patients treated with MF and 27.9 (SD 3.8) in the OAT mosaicplasty group.

Reoperations and additional surgical procedures during follow-up are outlined in Table 4.

**Table 4:** Reoperations and additional surgical procedures during follow-up

	MF (*n* = 11)	OAT mosaicplasty (*n* = 14)
Procedures	6	5
ACI	2	
OAT mosaicplasty	1	
Open wedge osteotomy		1
Removal of loose body	1	
Diagnostic arthroscopy/debridement	1	4
Scheduled to TKA	1	

*ACI* autologous chondrocyte implantation, *TKA* total knee arthroplasty

## Discussion

The main finding of the present study is that the long-term outcomes following MF and OAT mosaicplasty for treating focal chondral lesions of the knee are comparable. The evidence in this material is not sufficient to reject the study hypothesis that there is no difference between the two alternative treatments. However, the small number of included patients makes any firm conclusions regarding the hypothesis testing difficult. Due to less eligible patients for the study than expected, the duration of the inclusion period was extended up to 5 years. Still only 25 patients were enroled in the study. However, no patients were lost to follow-up.

Reoperations occurred in 6/11 patients (54 %) in the MF group and in 5/14 patients (36 %) in the OAT mosaicplasty group. Even though non-significant, all knees that underwent a second cartilage repair procedure (*n* = 3) or a total knee arthroplasty (*n* = 1) belonged to the MF group. It should also be noted that a significant reduction in extension force of the affected leg, compared to the unaffected, was found in the OAT mosaicplasty group, even though a mini-invasive arthrotomy was used when possible.

Both treatment groups reported significant improvement in Lysholm score and in several of the KOOS subscales from baseline to follow-up at 9.8 years. However, the mean Lysholm score and KOOS at follow-up were considerably lower than in the reference population [5, 35], which indicates that the long-term patient-reported outcomes are modest for both treatments. In addition, the wide confidence intervals indicate diversity among the patients, which however, is not an uncommon finding in long-term follow-up studies on cartilage repair [4, 45]. The unpredictability of these two cartilage repair methods has been found in standardized controlled animal studies as well [19].

To our knowledge, there are only two other clinical studies comparing MF and OAT mosaicplasty [12, 28]. In the only randomized trial, the OAT mosaicplasty-treated patients scored significantly higher on the ICRS outcome scores and Tegner scores compared to the MF-treated patients at a mean follow-up of 10.4 years [12]. Furthermore, the failure rate and the decrease in sports activity were significantly higher for the MF group. Although our study did not demonstrate any significant difference regarding reoperations, the trend was that reoperations occur more often in the MF group. However, comparison between the studies is difficult due to differences in study populations, surgical techniques and the use of other outcome measures. Gudas et al. included competitive or well-trained athletes, whereas the present study did not exclude non-athletes. Several studies indicate that both OAT mosaicplasty and MF provide favourable outcome in small lesions [4, 8, 25, 31, 34]. The fact that relatively small-sized lesions <2 cm^2^ were included and that lesions >4 cm^2^ were excluded in the Gudas study might in part explain the apparently better results at follow-up in that study compared to the present study. Another difference between these two studies is that in the Gudas study, all OAT mosaicplasty patients were treated arthroscopically, whereas in the current study an arthrotomy was performed in all mosaicplasty procedures.

In a recent retrospective, comparative study, Krych et al. [28] showed that both MF and OAT mosaicplasty-treated patients reported significant improvements in knee function and activity level at 5-year follow-up. No significant differences were detected between the two groups regarding knee function, but the mosaicplasty group maintained a superior level of activity compared to those treated with MF. The main findings of that study are in line with those of the present study, but the validity of the conclusions in the study of Krych et al. is limited by the study design, since it allows for selection bias. The unevenly distributed number of patients with previous cartilage surgery, and osteochondritis dissecans, should also be accounted for in the study by Krych et al.

There are few long-term follow-up studies following MF for treating chondral lesions of the knee. In a systematic review by Mithoefer et al. [32] only 5 studies reported a follow-up of 5 years or more, and the reports on the durability of the initial functional improvement were conflicting. The present study shows that functional improvement after MF is to be expected as long as 9.8 years after surgery.

The long-term outcome following OAT mosaicplasty in the present study supports the findings from other studies on OAT mosaicplasty, indicating acceptable long-term clinical outcome given the appropriate indication for surgery, a limitation being the defect size [12, 14, 41].

The main limitation of this study is the small number of included patients, which may lead to a false affirmation of the null hypothesis (type II error). On the other hand, the follow-up of 100 % for the main outcome (Lysholm score), and the high follow-up (88–100 %) and uniformity of comparable results between the two groups in the additional broad spectrum of outcome measures, strengthens the validity of the conclusion. Other limitations of the study are lack of a mid-term evaluation and the incompleteness of the preoperative strength measurements.

In the light of the limited information in current literature on the topic of long-term comparison between MF and OAT mosaicplasty, there is a need for further RCTs and a future cartilage repair registry in order to monitor and assess the cartilage repair procedures in use. The results from the current study might help the orthopaedic surgeon in the preoperative decision-making and in informing the patient what to expect concerning long-term outcome following these two cartilage repair techniques.

## Conclusion

At long-term follow-up, there were no significant differences between patients treated with MF and patients treated with OAT mosaicplasty in patient-reported outcomes, muscle strength or radiological outcome. Both MF-treated as well as OAT mosaicplasty-treated patients reported improved knee function compared to the preoperative level. However, compared to a reference population, inferior patient-reported knee function was found in both treatment groups at follow-up.
